# Structural Insights into the SARS-CoV-2 Spike Protein and Its Implications for Antibody Resistance

**DOI:** 10.3390/biom15111489

**Published:** 2025-10-22

**Authors:** Yuichiro Yamamoto, Kohji Noguchi

**Affiliations:** 1 Laboratory of Molecular Targeted Therapy, Faculty of Pharmaceutical Sciences, Tokyo University of Science, 6-3-1, Niijuku, Katsushika-ku, Tokyo 125-8585, Japan; yuichiro-yamamoto@rs.tus.ac.jp; 2 Department of Biochemistry and Cell Biology, National Institute of Infectious Diseases, Japan Institute for Health Security, 1-23-1, Toyama, Shinjuku-ku, Tokyo 162-8640, Japan

**Keywords:** SARS-CoV-2, antibody resistance, RBD, S2 subunit, bispecific antibody

## Abstract

The COVID-19 pandemic, caused by SARS-CoV-2, has profoundly affected global health and the economy. The emergence of variants with spike mutations, particularly within the receptor-binding domain (RBD), has reduced the efficacy of many neutralizing antibodies (nAbs), and recent variants, including KP.3 and other circulating strains, show partial escape from infection- or vaccine-induced immunity. To overcome this, developing broad-spectrum nAbs that target the conserved S2 subunit of the spike protein is crucial. Unlike the highly mutable RBD, the S2 region remains structurally conserved, providing a promising foundation for universal protection. Deeper insight into S2 structure and function, together with advances in bispecific antibody design, could facilitate the development of next-generation therapeutics resilient to viral evolution. This review examines the structural evolution of the SARS-CoV-2 spike, focusing on the therapeutic potential of S2-targeting antibodies and strategies to overcome antibody resistance.

## 1. Introduction

The spike (S) glycoprotein of severe acute respiratory syndrome coronavirus 2 (SARS-CoV-2) is essential for mediating viral entry into host cells, and it plays a central role in viral infectivity, host tropism, and immune recognition. The S protein is embedded in the viral envelope as a homotrimer, with each monomer comprising two functional subunits: S1 and S2 [1]. The S1 subunit (residues 1–685) mediates initial attachment to host cells and contains both the N-terminal domain (NTD) and the receptor-binding domain (RBD). This RBD directly engages the angiotensin-converting enzyme 2 (ACE2) receptor on host cells, facilitating viral attachment [1,2]. The S2 subunit (residues 686–1273) contains highly conserved structural elements including the fusion peptide (FP), stem helix (SH), and heptad repeats 1 and 2 (HR1 and HR2), which mediate the fusion of viral and host membranes [1,2,3]. Structural and functional studies have shown that the S protein undergoes major conformational rearrangements during receptor engagement. RBD binding in its “up” conformation to ACE2 initiates S1 subunit dissociation and triggers the S2 subunit’s transition to a postfusion conformation, driving membrane fusion [1,2,3]. These structural transitions—particularly in the RBD—are critical determinants of infectivity and immune evasion.

Accumulation of amino acid mutations in the SARS-CoV-2 S protein leads to substantial changes in its three-dimensional conformation and dynamics. These alterations can modulate epitope accessibility and antigenicity, ultimately affecting neutralizing antibody recognition and the efficacy of therapeutic antibodies [4,5,6]. Recent studies have also highlighted that the dynamic movement of the RBD—switching between “up” and “down” states—regulates antibody accessibility. Because the most potent neutralizing antibodies target the RBD, their structural classification provides insight into how spike conformational states influence antibody binding. Neutralizing antibodies (nAbs) targeting the SARS-CoV-2 RBD are generally classified into four classes based on their epitope and binding mode. Class 1 and 2 antibodies bind the receptor-binding motif (RBM) and block ACE2, with Class 1 preferring the RBD-up state and Class 2 recognizing both up and down conformations. Class 3 antibodies target a conserved site on the outer RBD surface, often showing broad sarbecovirus reactivity, while Class 4 antibodies bind a cryptic inner epitope exposed only in the RBD-up conformation [4,7]. Omicron subvariants shift this balance toward the “down” state, concealing epitopes targeted by Class 1 and 2 antibodies and thereby promoting immune escape [8,9].

Cryo-EM and functional analyses have revealed that the F486V and R493Q mutations in the RBD of SARS-CoV-2 Omicron subvariants play a pivotal role in modulating antibody escape and receptor engagement. The F486V substitution disrupts interactions with a hydrophobic pocket on ACE2 and significantly impairs the binding of Class 1 and 2 nAbs. In contrast, the R493Q reversion enhances ACE2 affinity and partially restores susceptibility to neutralization [10,11]. Moreover, S2 mutations—such as D796Y and L981F found in Omicron sublineages—have been shown to impact the global trimer architecture of the spike, allosterically altering RBD exposure and reducing susceptibility to Class 1 and 2 antibodies [12,13]. Therefore, coordinated mutations in both the RBD and S2 regions can induce allosteric changes that reshape the global architecture of the spike trimer, thereby further complicating antibody access and neutralization. These findings underscore the importance of structure-guided approaches in antibody drug development.

Vaccination has also played a pivotal role in mitigating SARS-CoV-2 infection and disease burden worldwide. COVID-19 vaccines elicit potent neutralizing antibody responses targeting the spike RBD, effectively blocking viral entry by preventing ACE2 engagement [14,15]. Vaccine-induced immunity wanes over time, and the emergence of immune escape variants has reduced neutralization potency [16,17]. Although booster doses and updated formulations have partially restored neutralization breadth, these limitations highlight the need for alternative or complementary therapeutic strategies [16,17,18].

Since the onset of the pandemic, numerous monoclonal nAbs have been developed, most of which target the RBD due to its key role in receptor binding and strong immunogenicity [19,20]. However, substitutions such as R346T, K417N, L452R, E484A, Q493R, and N501Y in the RBD have been shown to significantly impair or abolish the binding of many clinically approved nAbs. These mutations affect key contact residues within antibody epitopes, altering local conformation, electrostatics, or hydrophobicity, thereby reducing the antibody affinity and neutralization potency [21,22,23,24,25]. While the RBD is prone to novel mutations, the S2 subunit of the S protein is more conserved across SARS-CoV-2 variants and beta-coronaviruses. Notably, key conserved elements within the S2 region—including the FP, SH, HR1, and HR2—exhibit high sequence conservation, underscoring their potential as targets for broad-spectrum antibody therapeutics [26,27,28]. In fact, previous studies have demonstrated that certain monoclonal antibodies (mAbs) targeting the S2 subunit of the SARS-CoV-2 S protein possess broad neutralizing activity [29,30,31]. Recently, the anti-S2 antibody Cv2.3132 (targeting the HR2 region) neutralized Omicron subvariants BA.4.6, BQ.1.1, XBB.1, XBB.1.5, and the more recent JN.1 in vitro, with IC50 values ranging from 4.8 to 10.4 μg/mL [32].

Previous studies have shown that S2-targeting mAbs generally exhibit low neutralization potency in vitro, likely due to limited epitope accessibility or transient exposure of the S2 subunit during membrane fusion [33,34,35,36]. Additionally, the S2 subunit is immunologically subdominant in terms of neutralizing antibody responses, as most S2-binding mAbs isolated from SARS-CoV-2-infected individuals lack neutralizing activity [37,38,39,40]. The continuous accumulation of SARS-CoV-2 spike mutations has reduced the neutralization efficacy of both therapeutic and vaccine-induced antibodies, reflecting the ongoing antigenic drift of the virus—a process that continually alters antigenic sites—and posing a persistent challenge to maintaining both therapeutic efficacy and vaccine-derived protection. Several strategies have been proposed to overcome immune escape in RBD-targeting antibodies and the limited potency of S2-targeting antibodies. Antibody cocktail therapy, which targets multiple distinct epitopes, has potential value. It can prevent the emergence of resistant variants and offers the additional advantage of preventing the epitope escape of SARS-CoV-2 variants [41,42]. Nanobody-based strategies prevent immune escape by targeting cryptic epitopes that are inaccessible to conventional antibodies. Their multivalent formats enable the simultaneous recognition of distinct sites, reducing the impact of mutation-induced epitope loss or structural changes while enhancing stability and delivery efficiency [43,44]. Furthermore, another promising strategy involves the use of bispecific antibodies (bsAbs) that simultaneously target non-overlapping epitopes, which have demonstrated enhanced neutralization potency in vitro and conferred protection in animal models, indicating their potential to suppress immune escape [45,46,47]. BsAbs targeting two distinct epitopes within the RBD can neutralize many SARS-CoV-2 variants, but can also block the escape mutants generated by parental mAbs [45].

In this review, we first summarize the key structural features of representative RBD mutations that drive resistance to nAbs. We then examine the amino acid mutations in the S2 subunit identified in recent Omicron subvariants and their effects on antibody binding and neutralization. Finally, we highlight antibody engineering strategies—particularly bsAbs targeting both the RBD and S2—that aim to overcome these resistance mechanisms. Together, these insights underscore the importance of structure-guided antibody design for developing next-generation therapeutics capable of maintaining efficacy against evolving SARS-CoV-2 variants.

## 2. Structural Characteristics of RBD Mutations Associated with Antibody Resistance

**Figure 1:**
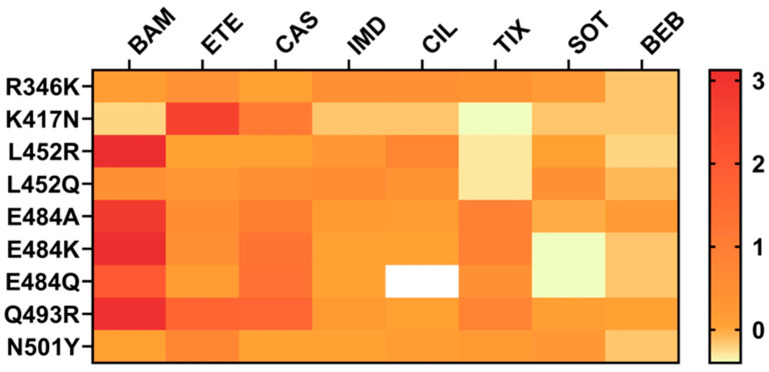
Fold change values in neutralization by single escape mutations against therapeutic mAbs. These values were compiled from multiple independent studies curated in the Stanford Coronavirus Resistance Database (CoV-RDB; https://covdb.stanford.edu). As experimental conditions (e.g., assay types, viral backbones, antibody formats) vary across studies, these values may not be directly comparable. Values are presented to highlight general resistance trends rather than precise quantitative differences. BAM: bamlanivimab, ETE: etesevimab, CAS: casirivimab, IMD: imdevimab, CIL: cilgavimab, TIX: tixagevimab, SOT: sotrovimab, BEB: bebtelovimab.

The S1 subunit of the SARS-CoV-2 S protein, consisting of the NTD and RBD, has been a major target for nAbs [19,20]. Specific amino acid substitutions—such as R346K, L452R, E484K, and Q493R—have been shown to confer varying degrees of resistance to Food and Drug Administration (FDA)-authorized therapeutic nAbs (Figure 1). Understanding the structural consequences of such mutations is essential for developing next-generation therapeutics. This section focuses on amino acid substitutions with high prevalence in the Stanford Coronavirus Resistance Database (CoV-RDB; https://covdb.stanford.edu, accessed on 3 September 2025) [48], which were selected for review. We highlight representative mutations associated with resistance to anti-RBD nAbs and their structural implications for both antigen and antibody.

*R346T and R346K*

The R346 residue within the RBD of the SARS-CoV-2 spike protein has emerged as a key mutational hotspot that affects immune escape and viral fitness, with R346T and R346K being the most frequently observed substitutions in circulating variants [49,50]. The R346T mutation has been identified in multiple Omicron sublineages including BA.4, BA.5, BA.2.75.2, BF.7, BQ.1.1, and XBB [21,51,52]. Structural analyses have shown that the R346T mutation significantly impairs the binding of therapeutic Class 3 nAbs targeting the RBD by disrupting critical hydrogen bond interactions at the antibody–epitope interface and inducing local conformational changes. Cryo-EM studies revealed that this mutation alters the antigenic surface of the RBD, thereby diminishing the binding affinity of broadly neutralizing antibodies such as S309 (sotrovimab) and AZD1061 (cilgavimab) [21,51]. Several studies have shown that R346T contributes to immune escape when present alongside other mutations such as K444T, F486S, and D1199N [51,53]. Its repeated emergence in different lineages reflects convergent evolution under antibody selective pressure [54]. In contrast, several studies have shown that the R346K substitution, despite occurring at the same immunologically critical site as R346T, has a limited impact on antibody-mediated neutralization, suggesting that it is structurally and antigenically more conservative [55].

*K417N*

The K417 residue lies outside the core ACE2-binding region (residues 437–508) on the side of the concave RBD surface [56]. Substitution of lysine at this position with asparagine (K417N) has been linked to enhanced resistance to therapeutic mAb neutralization [22,57,58,59]. The K417N mutation impairs a key contact site for VH3-53-derived nAbs by abolishing critical CDR-H3 interactions, thereby contributing to immune escape [22,58]. Conversely, mAb 222, a member of the VH3-53 family, retains neutralization potency against variants harboring K417N due to its unique structural features including minimal reliance on K417 contacts and compensatory interactions involving its somatically mutated light chain [60]. Furthermore, the K417N mutation introduces an immune escape mechanism by abolishing a buried interfacial salt bridge, which impairs the binding of nAbs such as COVA2-04, COVA2-07, and CB6 [61,62,63]. Although some nAbs tolerate the K417N mutation alone, their activity is markedly reduced when this substitution is combined with additional Omicron RBD mutations, such as S477N, Q493R, G496S, Q498R, N501Y, and Y505H, as K417N alters a critical salt bridge between Lys417 in the RBD and a negatively charged residue in the antibody, thereby impairing antibody binding and contributing to immune escape [64].

*L452R*

The L452R mutation has emerged in the RBD of various SARS-CoV-2 variants, including Omicron sublineages such as BA.4, BA.5, and BQ.1.1, illustrating a clear example of convergent evolution [65]. Residue L452, which is implicated in resistance to Class 2 and 3 nAbs, interferes with the binding of antibodies that target nearby epitopes on the RBD [11,66]. L452R has been reported to diminish the binding of several mAbs, likely due to steric hindrance and altered electrostatic interactions. For example, structural modeling further suggests that the increased resistance of L452R to AZD1061 arises from steric clashes at the antibody–spike interface [23,67]. Lambda (L452Q/F490S) and Omicron BA.2.86 (L452W) show markedly reduced neutralization by VH1-69-class antibodies targeting the RBD core epitope. Structural analysis indicates that these substitutions disrupt the hydrophobic patch formed by RBD residues L452, F490, and L492, which engages the hydrophobic CDRH2 of VH1-69 antibodies. In particular, the combination of L452Q and F490S in Lambda or the bulky L452W substitution in BA.2.86 more effectively interferes with this hydrophobic interaction, leading to escape from neutralization [68].

*E484K*

The E484K mutation involves the substitution of a negatively charged glutamate with a positively charged lysine residue at position 484. Several studies have shown that this mutation can introduce new electrostatic interactions with ACE2, particularly forming a potential salt bridge with Glu35 or 75 on ACE2, thereby enhancing binding affinity [69,70,71]. The E484K mutation can reduce the binding of Class 2 nAbs by altering multiple structural features of the antibody–RBD interface. It disrupts favorable electrostatic interactions, exemplified by the loss of the salt bridge between E484 and the CDR H3 residue R103 in antibody P2B4 [72], and it can perturb local hydrophobic contacts around E484 that contribute to the binding of antibodies such as C121 and C144 [73]. The E484K present in Omicron subvariants such as BA.2.86, JN.1, and KP.3 disrupts the salt bridge between E484 and R59 in the CDR-H2 of the Class 2 antibody NT-108, leading to a loss of neutralization. Structural analysis suggests that the lysine side chain also alters the local interaction network at the antibody–RBD interface, collectively reducing the ACE2-binding inhibitory activity of NT-108 [74]. In addition, structural and escape-mutation analyses of CSW2-1353 indicate that substitutions at E484, such as E484K or E484A, lead to the loss of interactions with its CDR H2 and contribute to neutralization escape [75]. Furthermore, E484K contributes to immune escape when combined with mutations such as K417N and N501Y [76], and the analogous E484A found in the Omicron variant can reasonably be speculated to exhibit similar resistance profiles [74,77].

*Q493R*

The Q493R substitution in the SARS-CoV-2 Omicron variant is implicated in enhanced receptor engagement and immune evasion. Several studies have shown that the Q493R and Q498R mutations introduce new salt bridges with ACE2 residues Glu35 and Glu38, replacing hydrogen bonds formed with the Wuhan-Hu-1 RBD and thereby strengthening the electrostatic interactions with ACE2 [25,78,79]. Cryo-EM and crystallographic analyses demonstrated that Q493R induces steric hindrance and perturbs electrostatic interactions, thereby disrupting the binding of Class 1–2 antibodies, including REGN10933, LY-CoV016, and LY-CoV555, which leads to a marked reduction in neutralization potency [25,78]. In addition, Q493R, together with neighboring substitutions such as G496S and Y505H, is associated with local conformational changes in the RBM that introduce steric clashes and perturb electrostatic complementarity at the antibody–RBD interface, further weakening the binding of these therapeutic antibodies [25]. Although REGN10987 (Class 3) is primarily impaired by other Omicron mutations such as G446S, Q493R in combination with adjacent substitutions contributes to the extensive immune escape phenotype observed in Omicron [78,80]. The reversion mutation R493Q, as observed in the Omicron subvariant BA.2.75, partially restores neutralization sensitivity compared with the Q493R-containing variants [81]. Structural studies revealed that Gln493 forms favorable hydrogen bonds with ACE2 residues Lys31 and Glu35, alleviating the electrostatic repulsion introduced by Arg493 [82]. Consistently, a comparison of neutralization by therapeutic mAbs across different classes—including COV2-2196 (tixagevimab), S2K146, and XGv282—revealed that BA.2-R493Q exhibited lower resistance than XBB and XBB.1 [21], underscoring the critical role of residue 493 in immune escape.

*N501Y*

The N501Y mutation, which was observed in various lineages belonging to the Alpha variant and is also present in Omicron variants, contributes to enhanced ACE2 binding—facilitating viral entry—and plays a role in the increased transmissibility and immune escape [59,76,83,84,85]. Structural analyses have shown that the N501Y substitution enhances ACE2 binding by introducing two new hydrogen bonds between Y501 and ACE2 residues D38 and K353, and by establishing a strong π–π stacking interaction between the aromatic ring of Y501 and Y41 of ACE2 [61,86]. These additional interactions stabilize the RBD–ACE2 interface, contributing to the higher binding affinity and a more stable spike–ACE2 complex. Structural and functional analyses demonstrate that the N501Y mutation, located near the Class 1 antibody interface, has limited impact on the neutralization potency of therapeutic antibodies—including those from Classes 1, 2, and 3 such as REGN10933, REGN10987, and S309—as it does not significantly disrupt their respective epitope contacts, indicating that N501Y alone does not constitute a major antibody escape mutation [9,59]. Furthermore, computational structural analyses revealed that N501Y selectively alters antibody binding. For CB6 (Class 1, LY-CoV016), the hydrogen-bond network in CR1 remained intact, but the hydrophobic cluster in CR3 (Y32_l_, Y92_l_, Y505, R403) was disrupted, leading to a reduced contact area, water infiltration, and weakened binding affinity with a several-fold drop in neutralization potency. In contrast, CR3022 (Class 4) showed no appreciable change, while H014 (Class 4) exhibited slightly enhanced binding due to local conformational rearrangements that strengthen non-covalent interactions [87]. Together, these findings indicate that the N501Y substitution alone exerts only a limited impact on neutralizing antibody resistance. While N501Y alone imparts limited resistance to nAbs, the presence of co-mutations such as E484K and K417N markedly enhances immune escape. These findings underscore the importance of evaluating N501Y within the broader mutational context of the RBD [88].

## 3. S2 Mutations and Antibody Resistance in Omicron Variants

**Figure 2:**
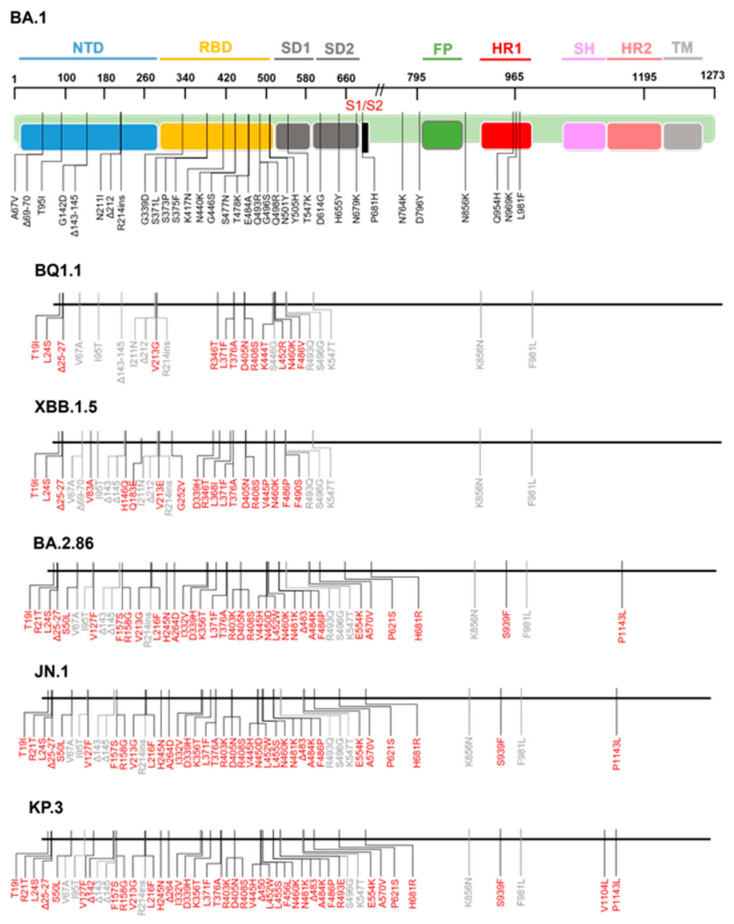
The mutational landscape of the Omicron spike protein. The amino acid modifications are indicated in comparison to the reference Omicron-BA.1 sequence. Consensus sequences of the spike protein were built using CoV-RDB. Amino acid substitutions are shown relative to BA.1. Residues that differ from BA.1 are highlighted in red. For positions where BA.1 already carries a substitution, the change is indicated relative to BA.1.

**Figure 3:**
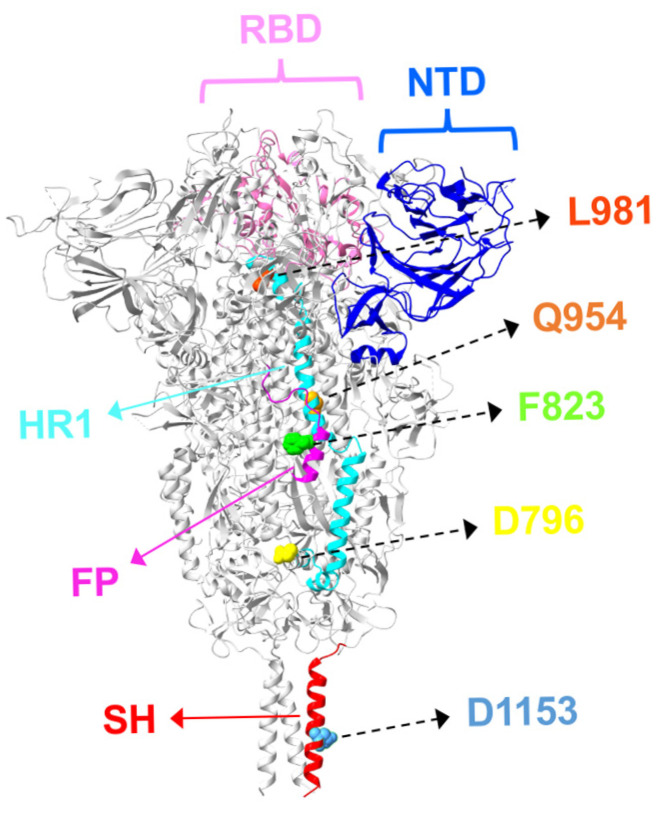
Structural overview of S2 mutations on antibody resistance in SARS-CoV-2. The structure of the SARS-CoV-2 spike trimer was obtained from the Protein Data Bank (PDB ID: 6XR8). Positions of key S2 subunit mutations associated with antibody resistance are displayed as spheres and color-coded: D796 (yellow), F823 (lime), Q954 (orange), L981 (orange-red), D1153 (light blue), NTD (residues 14–305, blue), RBD (residues 333–527, pink), FP (residues 816–835, magenta), HR1 (residues 906–986, cyan), and SH (residues 1137–1162, red).

Recent Omicron sublineages have underscored the significance of amino acid substitutions in the S2 subunit (Figure 2), which have been linked to enhanced fusogenicity, immune evasion, and reduced susceptibility to nAbs—including those that do not directly target the S2 region. This section highlights key S2 mutations and examines their structural implications for antibody resistance (Figure 3).

### 3.1. Fusion Peptide Mutations and Antibody Resistance

F823Y is observed at a very low frequency among SARS-CoV-2 genomes on GISAID, yet this substitution reduces the binding of the FP-targeting antibodies COV44-62 and COV44-79, thereby conferring neutralization resistance. The F823Y substitution confers resistance to FP-targeting antibodies by introducing a hydroxyl group that becomes buried in a hydrophobic pocket, creating unfavorable interactions that destabilize antibody binding [89].

### 3.2. HR1/HR2 Region Mutations and Antibody Resistance

Analysis of the impact of S2 subunit mutations on neutralization sensitivity showed that certain S2 variants, particularly D796Y and L981F, exhibited reduced sensitivity to NTD-targeting antibodies (COVA2-17, 4A8) and an RBD-targeting antibody (S309), whereas sensitivity to S2-specific antibodies (CC40.8, CV3.25) remained unchanged. Mechanistically, S2 mutations restrict the RBD “up” transition and epitope exposure, thereby reducing neutralization by NTD- and RBD-targeting antibodies, as supported by delayed neutralization kinetics and reduced maximal neutralization. Multiple mutations in the S2 region have been shown to impair the binding activity of IGHV1-69/IGKV3-11 antibodies. In particular, D950N, Q954H, T961F, V987C, Q1005R, and Q1010W reduced antibody binding. Notably, the Q954H mutation is fixed in naturally occurring variants such as BA.2.86 and JN.1. These spatially distributed mutations appear to modulate antibody recognition not by altering direct epitope residues, but rather by perturbing spike stability and conformational dynamics [90]. The residue D1199 lies on a solvent-accessible surface near the transmembrane domain, where its strong negative potential repels the membrane and stabilizes the spike upright. The D1199N substitution reduces this repulsion, tilting the spike and altering receptor utilization. These findings provide a mechanistic basis for receptor usage changes and antibody evasion in subvariants such as BQ.1 and BQ.1.1 [51].

### 3.3. Stem-Helix Mutations and Antibody Resistance

The S2 SH-targeting antibodies CC9.104 and CC67.105 exhibit broad neutralizing activity against diverse SARS-related coronaviruses. Deep mutational scanning revealed that the escape sites for both antibodies are clustered within the S2 SH region. The epitope of CC67.105 is centered on residues D1146, D1153, and F1156 with substitutions at these positions resulting in complete loss of neutralization. Moreover, even in SARS-CoV-2, mutational diversity can facilitate escape from both antibodies; notably, the Omicron sublineages BA.2.46 and BA.2.59 harbor the D1153Y substitution, which confers near-complete escape [91].

Overall, the continued evolution of Omicron subvariants has led to the emergence of mutations even in the previously stable S2 subunit, enhancing infectivity and resistance to nAbs. Such layered immune escape highlights the need to reassess current vaccines and therapies. Furthermore, antibody resistance mechanisms involving S2 mutations remain comparatively underexplored, emphasizing the need for further detailed investigations in this field.

## 4. Optimizing Antibody Therapeutics with BsAbs for Breadth and Resistance

Recent Omicron sublineages such as KP.3 and XEC exhibit broad resistance to therapeutic mAbs, with previously authorized antibodies (AZD7442, LY-CoV1404, VYD22) losing activity and S309 showing only limited efficacy or failure against newer lineages [92,93,94,95]; even the most recent pre-exposure prophylaxis (PrEP) antibody such as VYD222 displays lineage-specific gaps, and the emergent JN.1 progeny (KP.3.1.1 and XEC) further increase antibody evasion [96,97], challenging antibody-based interventions. In this context, this section reviews bsAbs—which integrate two non-overlapping specificities within a single molecule to restore breadth—and outlines design principles across RBD–RBD, RBD–NTD, and RBD–S2 designs.

### 4.1. RBD–RBD-Targeting Distinct Epitopes

RBD–RBD bsAbs are the most extensively studied bispecifics against SARS-CoV-2, showing superior potency and breadth compared with parental mAbs or cocktails across both pre-Omicron and Omicron sublineages.

Key advantages of bsAbs include enhanced neutralization of resistant variants, suppression of escape mutants, and the ability to overcome resistance to therapeutic antibodies. Among these, enhanced neutralization has been consistently demonstrated in representative studies. For instance, GW01-REGN10989 (G9) neutralized tested escape mutants, including Omicron, and remained effective against variants resistant to GW01 or REGN10989 alone as well as other NAb-escape mutants [98]. Similarly, 14-H-06 exhibited superior breadth and potency, retaining efficacy against resistant mutants including K444R, E484A, E484K/N501Y, and K417N/E484K through enhanced inter-spike crosslinking [99]. Notably, ISH0339 restored blocking activity against BQ.1.1 harboring the R346T mutation, even when its parental antibody lost efficacy [100]. Furthermore, several studies have shown that bsAbs can effectively suppress the emergence of escape mutants. In bsAb15, no dominant RBD mutations were observed, whereas escape variants like E484A, N460S, and V407A readily emerged under the selective pressure of parental antibodies or their cocktail. These results indicate that bsAbs impose weaker selective pressure but retain potent neutralizing activity [101]. Sybodies are synthetic single-domain antibodies generated in vitro to mimic camelid nanobodies and bind target proteins with high affinity. As another example, Sb#15–Sb#68 sybody fusion showed strong resistance to immune escape, effectively blocking resistant variants. In particular, GS4 not only enhanced neutralization, but also mitigated the appearance of resistant mutants, whereas single sybodies readily allowed Q493R or P384H mutants to emerge [102]. Finally, a representative study demonstrated that bsAbs can overcome resistance to therapeutic antibodies that lost activity against emerging variants. Ly-CoV1404-based bsAbs restored neutralizing activity against resistant variants, including Omicron XBB.1, SARS-CoV, and WIV1, and exhibited cooperative effects beyond antibody cocktails, resulting in improved potency [103].

At the structural level, Cryo-EM analyses have revealed convergent mechanisms that account for the superior breadth and potency of RBD–RBD bsAbs. Representative structures show that certain bsAbs engage the spike in the three-RBD-up conformation and achieve multivalent binding. For example, K202.B binds only fully open spikes and simultaneously engages non-overlapping RBM and core epitopes with its Fab and scFv, increasing the buried surface area; in contrast, bsAb1 binds spikes with all three RBD-up states, but its Fab and scFv target overlapping epitopes. Notably, both K202.B and bsAb1 stabilize the spike in a fully open three-RBD-up state, a “locking” effect that provides the structural basis for their potent neutralizing activity [104,105]. G7-Fc and GW01–16L9 (FD01) stabilized the spike trimer in a fully open three-RBD-up state through Fc-mediated cross-linking and complementary epitope recognition, thereby blocking ACE2 binding, a mechanism that exemplifies how RBD–RBD bsAbs enhance neutralization [106,107]. bn03 engages both an exposed epitope and a cryptic site at the trimer interface, promoting a wide-open RBD conformation and destabilizing the spike trimer, which explains its broad and potent neutralization [108]. Alternatively, 123-364 scDb is suggested to facilitate inter-spike cross-linking, thereby promoting extensive RBD opening and exposing a cryptic epitope. This conformational change is suggested to enable binding to otherwise inaccessible sites, thereby restoring neutralization against immune-evasive variants [109].

### 4.2. RBD–NTD-Targeting Dual Epitopes

Few studies have described RBD–NTD bsAbs, but they establish a proof-of-concept for the broad neutralization of resistant SARS-CoV-2 variants. In a dual variable domain (DVD-Ig) format, these bsAbs mediated the unique cross-linking of adjacent spikes and achieved superior potency; notably, CV1206_521_GS was over 100-fold more potent than a cocktail of its constituent antibodies. Importantly, they also retained robust activity against pre-Omicron variants, with CV503_664_EL maintaining potency against Beta despite reduced efficacy of both parents [47]. More recent studies have further demonstrated that RBD–NTD bsAbs sustain higher neutralizing potency than parental antibodies or cocktails including against emerging Omicron subvariants (e.g., BA.2.86, JN.1, and KP.3) [110,111].

### 4.3. Dual-Epitope-Targeting BsAbs: RBD–S2 and ACE2–S2

RBD–S2 bsAbs leverage the capacity to simultaneously target the RBD and the S2 subunit of the SARS-CoV-2 spike, thereby synergistically blocking viral attachment and membrane fusion. Several constructs have further demonstrated therapeutic efficacy in vivo, reducing viral loads and lung pathology in infected animals (Table 1).

In early studies of SARS-CoV-2, a representative case was Bi-Nab_35B5-47D10_, an IgG-like construct combining the RBD-directed mAb 35B5 with the S2-specific 47D10. While each parental antibody displayed limited breadth, their combination enabled the simultaneous engagement of distinct epitopes, resulting in enhanced neutralization across multiple variants, including Delta and Omicron sublineages, compared with the parental mAbs and cocktails. This dual inhibition mechanism highlights how RBD–S2 targeting can overcome the limitations of single-site antibodies [112]. Another notable example is K203.A, an IgG4-(scFv)_2_ bsAb constructed from an RBD-specific mAb and an FP-specific mAb in the S2 subunit. Incorporation of the FP arm enhanced potency against SARS-CoV-2 variants that were poorly neutralized by the parental RBD-specific mAb, and in vivo administration in K18-hACE2-transgenic mice reduced lung viral loads and lung pathology. Furthermore, SPR analysis confirmed the dual-targeting potential of K203.A to simultaneously engage both the SARS-CoV-2 RBD and FP. This strategy underscores the therapeutic potential of targeting receptor binding and membrane fusion in tandem [107]. Additional strategies exploit non-neutralizing or weakly neutralizing antibodies that gain efficacy through bispecific engineering. Bis3, generated from a non-neutralizing RBD-specific mAb (CvMab-6) and a weakly neutralizing S2-specific mAb (CvMab-62), restored neutralizing activity against SARS-CoV-2 variants. In addition, a bsAb derived from LY-CoV1404 (Bebtelovimab) and CvMab-62 overcame the LY-CoV1404 resistance observed in the BQ.1.1. Importantly, Bis3 inhibited spike-mediated cell–cell fusion, directly demonstrating a fusion-inhibitory mechanism [114]. A further representative example was F-S309 + S2P6 (with S2P6 targeting the SH region) and related constructs, which retained activity against immune escape Omicron sublineages and showed superior lung protection in vivo [115]. Finally, an alternative strategy has been the development of bsAbs combining the anti-ACE2 antibody with anti-spike antibodies. H11B11_S2P6 and related constructs achieved broad neutralization against SARS-CoV-2 variants and SARS-CoV. IgG-scFv formats outperformed Knob-into-Hole designs in bsAbs that combined H11B11 with anti-spike antibodies, highlighting the role of Fc engineering and linker flexibility in optimizing ACE2-based bsAbs [116].

In summary, bsAbs represent a structurally informed strategy to counter SARS-CoV-2 immune escape. RBD–RBD bsAbs confer superior potency and breadth, while the RBD–S2 and RBD–NTD formats extend protection by combining receptor blockade with fusion inhibition or by stabilizing multivalent spike conformations. Structural mechanisms such as three-RBD-up stabilization, Fc-mediated cross-linking, and dual recognition of exposed and cryptic epitopes underpin their enhanced activity. With continued optimization of Fc design, linker flexibility, and in vivo performance, bsAbs stand as promising next-generation therapeutics with potential relevance for future coronavirus preparedness. However, cryo-EM data of RBD–S2 bsAbs in complex with the spike trimer are currently lacking, and the precise binding mode remains undefined. Future structural studies will be required to elucidate their mechanistic basis.

## 5. Future Directions

BsAbs exhibit greater manufacturing complexity than conventional mAbs. Their intricate architectures, such as asymmetric IgG-scFv or CrossMAb formats, can result in chain mispairing and product heterogeneity, complicating downstream chemistry, manufacturing, and control (CMC) processes like purification and quality control. To ensure proper assembly and stability, engineering strategies such as heterodimerization or common light chain designs are often implemented. These processes increase the production costs and analytical burdens in large-scale manufacturing. Nevertheless, advances in protein engineering, expression systems, and purification technologies are gradually overcoming these challenges, supporting the feasibility of bsAbs as scalable therapeutic and discovery platforms [117].

## 6. Conclusions

Numerous escape mutations have occurred in the SARS-CoV-2 spike, compromising the efficacy of therapeutic antibodies. This vulnerability to immune-evasive variants highlights the need for complementary strategies. BsAbs, by integrating dual-epitope recognition, enhance potency, breadth, and resistance suppression beyond parental mAbs or cocktails. These advances position bsAbs as promising next-generation therapeutics, warranting further structural and clinical investigation.

## Figures and Tables

**Table 1:** Representative bispecific antibodies targeting the S2 subunit of SARS-CoV-2.

Name	Format	Parent Antibodies	IC50 Pseudotyped Virus (nM)	IC50 Authentic Virus (nM)	Protective Efficacy	Ref.
Bi-Nab_35B5-47D10_, Bi-Nab_47D10-35B5_	IgG-(scFv)_2_	anti-RBD: 35B5 anti-S2: 47D10	Bi-Nab_35B5-47D10_ WT: 0.046, Alpha: 0.038, Beta: 0.36, Delta: 0.079, Kappa: 0.065, BA.1: 0.15, BA.2: 0.67 Bi-Nab_47D10-35B5_ WT: 0.12, Alpha: 0.083, Beta: 0.78, Delta: 0.81, Kappa: 0.052, BA.1: 1.52, BA.2: 2.88	n.a.	n.a.	[112]
K203.A	IgG4-(scFv)_2_	anti-RBD: K102.1 anti-S2 (FP): K107.1	D614G: 0.42 ± 0.02, Alpha: 0.33 ± 0.02, Beta: 1.29 ± 0.11, Gamma: 1.11 ± 0.10, Kappa: 5.46 ± 0.31	WT: n.d., Delta: n.d. (More potent than K102.1)	K203.A reduced viral loads and alleviated lung pathology in hACE2-TG mice (Delta variant).	[113]
Bis3, Bis-Beb	IgG-scFv	anti-RBD: CvMab-6, Bebtelovimab anti-S2: CvMab-62	Bis3 WT: 6.1, Alpha: 36.6, Delta: 141, BA.1:89 Bis-Beb BA.5.2: 1.6 × 10^−3^, K444T-BA.5.2: 2.8	Bis3 WT: 24.9, Alpha: 5.6, Delta: 163, BA.1: 13.1 Bis-Beb BA.5.2.1: 2.0 × 10^−2^, BQ.1.1: 2.6	n.a.	[114]
F-S2P6 + S309, F-S309 + S2P6	IgG-scFv	anti-RBD: S309 anti-S2 (SH): S2P6	F-S2P6 + S309 * WT: 0.056, Beta: 0.15, Delta: 0.28, BA.2: 0.58, BA.5: 0.62, XBB: 0.18 F-S309 + S2P6 * WT: 0.044, Beta: 0.078, Delta: 0.24, BA.2: 0.18, BA.5: 0.47, XBB: 0.16	n.a.	F-S2P6 + S309 and F-S309 + S2P6 reduced pulmonary viral loads in SARS-CoV-2-infected mice (XBB.1.16 subvariant).	[115]
H11B11_S2P6 S2P6_H11B11	Knob-into-Hole	anti-ACE2: H11B11 anti-S2 (SH): S2P6	H11B11_S2P6 ** WT: 0.99, BA.2: 1.15, BA.5: 1.53, XBB.1.5: 1.0 SARS-CoV: 1.61 S2P6_ H11B11 ** WT: 14.65, BA.2: 14.13, BA.5: 14.87, XBB.1.5: 24.17 SARS-CoV: 12.47	n.a.	n.a.	[116]

n.d., not determined. n.a., not available. * IC_50_ values are reported in ng/mL as originally described (Ref. [115]). Unless otherwise specified, approximate molar concentrations (nM) were calculated by the authors assuming a molecular weight of 180 kDa for IgG–scFv antibodies. Approximate estimates are not reported in the original publication; they are provided only for unit harmonization. ** IC_50_ values are reported in µg/mL as originally described (Ref. [116]). Unless otherwise specified, approximate molar concentrations (nM) were calculated by the authors assuming a molecular weight of 150 kDa for a typical IgG. Approximate estimates are not reported in the original publication; they are provided only for unit harmonization.

## Data Availability

No new data were created or analyzed in this study. Data sharing is not applicable to this article.
